# A comprehensive meta-analysis of genetic associations between five key SNPs and colorectal cancer risk

**DOI:** 10.18632/oncotarget.12154

**Published:** 2016-09-21

**Authors:** Yi Hong, Guoying Wu, Wei Li, Dahai Liu, Kan He

**Affiliations:** ^1^ Center for Stem Cell and Translational Medicine, School of Life Sciences, Anhui University, Hefei City, Anhui 230601, P. R. China; ^2^ Department of Biostatistics, School of Life Sciences, Anhui University, Hefei City, Anhui 230601, P. R. China

**Keywords:** colorectal cancer, GWAS, single nucleotide polymorphism, meta-analysis

## Abstract

Genome-wide association studies (GWAS) on colorectal cancer (CRC) have identified dozens of single nucleotide polymorphisms (SNPs) in more than 19 independent loci associated with CRC. Due to the heterogeneity of the studied subjects and the contrary results, it is challenging to verify the certainty of the association between these loci and CRC.

We conducted a critical review of the published studies of SNPs associated with CRC. Five most frequently reported SNPs, which are rs6983267/8q24.21, rs4939827/18q21.1, rs10795668/10p14, rs4444235/14q22.2 and rs4779584/ 15q13.3, were selected for the current study from the qualified studies. Then meta-analyses based on larger sample sizes with average of 33,000 CRC cases and 34,000 controls were performed to assess the association between SNPs and CRC risk. Heterogeneity among studies and publication bias were assessed by the *χ^2^*-based Q statistic test Begg's funnel plot or Egger's test, respectively.

Our meta-analysis confirmed significant associations of the five SNPs with CRC risk under different genetic models. Two risk variants at rs6983267 {Odds Ratio (OR) 1.388, 95% Confidence Interval (CI) 1.180-1.8633} and rs10795668 (OR 1.323, 95% CI 1.062-1.648) had the highest ORs in homogeneous model. While ORs of the other three variants at rs4939827 {OR 1.298, 95% CI 1.135-1.483}, rs4779584 (OR 1.261, 95% CI 1.146-1.386) and rs4444235 (OR 1.160, 95% CI 1.106-1.216) were also statistically significant. Sensitivity analyses and publication bias assessment indicated the robust stability and reliability of the results.

## INTRODUCTION

Colorectal cancer (CRC) is a leading cause of cancer and cancer deaths in the Western countries [1]. In many developing countries in Asia, the incidence of CRC is rising [2] as diet and lifestyle change.

CRC, as other complex diseases, is caused by both genetic and environmental factors. Twin studies have shown that inherited genetic factors contribute approximately 35% of the disease etiology [3, 4]. For the past decades, linkage studies on multi-case families have identified a number of rare mutations in highly penetrant genes (e.g. FAP, HNPCC, DNA mismatch repair (*MMR*) gene). Mutations in those genes are responsible for less than 5% of cases in the pathogenesis of CRC [5]; While, mildly or moderately penetrant genes could explain about 8.3% of etiology in cases with familial aggregation [6, 7]. It is expected that low-risk variants likely explain the remaining proportion of inherited susceptibility [5]. The candidate gene screening at some susceptibility loci such as (*MTHFR*) [8, 9] and cyclin D1 [10, 11], as well as the genome-wide expression studies [12, 13] were reported to identify associations with colorectal tumorigenesis. However, the replication and functional studies are needed to support the findings.

Recently association studies, directly comparing the frequencies of genetic variants between large series of affected cases and unrelated controls, are now considered to be more appropriate than linkage studies for the identification of susceptibility loci for complex diseases including CRC [14]. Over the past decade, with the resources of human genome and high-throughput platforms, Genome-wide association studies (GWAS) provide a powerful new approach allowing the scanning of the entire genome for association with disease to identify common, low-penetrance susceptibility loci without prior knowledge of biological function. GWAS have identified a number of loci that increase the risk of developing CRC. Early GWAS conducted in populations of European ancestry living in the United Kingdom (UK) and Canada have been published for the identification of 11 well-replicated disease loci, of which most were not previously suspected to be related to CRC. These variants map to 8q24.21 (rs6983267), 8q23.3 (rs16892766, EIF3H), 9q24 (rs719725), 10p14 (rs10795668), 11q23 (rs3802842), 14q22.2 (rs4444235, BMP4), 15q13.3 (rs4779584), 16q22.1 (rs9929218, CDH1), 18q21.1 (rs4939827, SMAD7), 19q13.1 (rs10411210, RHPN2) and 20p12.3 (rs961253) [15, 16]. The proportions of the familial and population risks explained by the published loci are small, thus a great deal of work is needed to understand the biological mechanisms underlying these associations. However, individual GWAS require large sample sizes to account for the inflated Type I error resulting from the very high number of case-control comparisons and to detect effect sizes that are expected to be small. Therefore, pooled data analysis can enhance the statistical power of the study. It enables us to summarize available evidence from larger sample sizes.

To date, over 40 loci have been reported to be associated with CRC risk, among which 19 Single nucleotide polymorphisms (SNPs) (Table 1) were reported at least twice by 2 or more study groups. Five SNPs at loci 10p14, 14q22.2, 15q13.3, 18q21.1 and 8q24.21 were replicated at least 3 times in publications [16-30]. However, the results from past studies on these SNPs are either controversial or inconclusive, such as rs10795668 and rs4779584 with some studies supporting a significant association, whereas others finding no association or an association in opposite direction. Thus, we performed an up to date meta-analysis to more precisely characterize the association between these 5 SNPs and CRC on a larger sample sizes.

**Table 1:** SNPs frequently reported to be associated with CRC risk

SNPs*	Chr/nearest Gene	Position	Reference
rs10411210	19q13.1/RHPN2	38224140	17,18
rs10774214	12p13.32/CCND2	4238613	10,22
**rs10795668**	**10p14**	**8741225**	15,18,20
rs12953717	18q21.1/SMAD7	44707927	13,16
rs16892766	8q23.3/EIF3H	117699995	15,18
rs1862748	16q22/CDH1	67390444	17,21
rs2423279	20p12.3/HAO1	7760350	10,22
rs3802842	11q23	110676919	16,18
**rs4444235**	**14q22.2/BMP4**	**53480669**	17,18,24
**rs4779584**	**15q13.3/GREM1**	**30782048**	15,18,21
rs4925386	20q13.33/LAMA5	60354439	12,18
**rs4939827**	**18q21.1/SMAD7**	**44707461**	13,15,16,18
rs5934683	Xp22.2/SHROOM2	9711474	10,25
rs647161	5q31.1/PITX1	134526991	10,22
**rs6983267**	**8q24.21/POU5F1, MYC**	**128482487**	11,14,15,18,19,24
rs7136702	12q13.13/LARP4	49166483	12,25
rs7229639	18q21.1/SMAD7	44704974	22,23
rs961253	20p12.3/BMP2	6352281	17,18
rs9929218	16q22.1/CDH1	67378447	17,18

* 5 SNPs in **bold** were selected for further analysis.

## RESULTS AND DISCUSSION

### Five most frequently reported SNPs were selected

Thirty relevant articles of CRC GWAS were identified after searching GWAS Central and PubMed. There were 149 individual SNPs reported to be associated with CRC risk. 19 of them were reported at least twice by different studies, 5 SNPs among which were most frequently reported: rs6983267, rs4939827, rs10795668, rs4444235 and rs4779584 (Table 1).

### Characteristics of included studies

**Figure 1:**
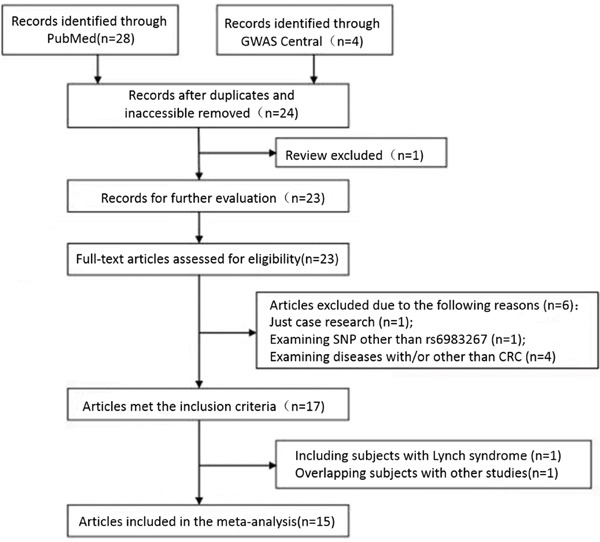
Flow chart of study selection on rs6983267

As shown in Figure 1, 32 potentially relevant articles were identified. 17 of these 32 articles met the inclusion criteria. However, one study including patients with Lynch syndrome and one study containing overlapping participants with other included studies were excluded after further examination. Finally, as a publication has more than one cohort in the study while another 3 have more than one population, a total of 15 publications with 24 data sets comprising 26,322 cases and 25,157 controls were included in the meta-analysis (Table 2). The allele frequencies of rs6983267 in controls conformed to Hardy-Weinberg equilibrium for all included studies (Supplementary Table S1).

**Table 2:** Characteristics of studies on the association between rs6938267 and CRC risk included in the meta-analysis

First author	Year	Ethnicity (Population)	Number	
			Case	Control
Real LM	2014	Spanish	500	801
Haerian MS	2014	Iranian	380	335
Li FX	2012	Southern Chinese	229	267
Thean LF	2012	Chinese	991	993
Tuupanen S	2008	Finnish	996	1012
Daraei A	2012	Iranian	115	120
Cui R	2011	Japanese	6167	4494
He J	2011	European American	1171	1534
He J	2011	African American	382	510
He J	2011	Native Hawaiian	323	472
He J	2011	Japanese American	1042	1426
He J	2011	Latino	393	524
Kupfer SS	2010	European American	399	367
Kupfer SS	2010	African American	795	985
von Holst S	2010	Swedish	1786	1749
Matsuo k	2009	Japanese	481	962
Middeldorp A	2009	Dutch	995	1340
Kupfer SS	2009	European Americans	288	202
Kupfer SS	2009	African Americans	281	237
Curtin K	2009	UK	654	621
Tomlinson I (A)	2007	UK	620	960
Tomlinson I (B)	2007	UK	4361	3752
Tomlinson I (C)	2007	UK	1901	1079
Tomlinson I (D)	2007	UK	1072	415

**Figure 2:**
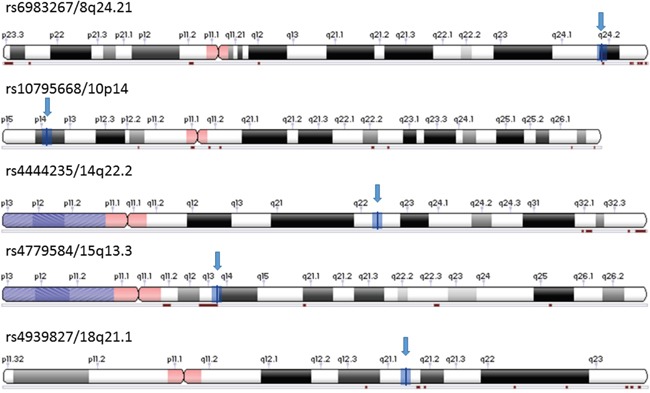
Location of five SNPs selected for meta-analysis Adopted from NCBI Variation Viewer 1.5.

The studies were included for the other 4 SNPs (Supplementary Table S2-S5) based on the same criteria showing in material and methods. The numbers of studies included for all 5 SNPs were listed in Table 3, and their locations on chromosomes are shown in Figure 2.

**Table 3:** Search results of studies on 5SNPs included in meta-analysis

SNP	Location/gene(nearest)	No. of publication after reviews excluded	No. of publication included in meta-analysis
rs6983267	8q24.21/POU5F1, MYC	32	15
rs4939827	18q21.1/SMAD7	23	12
rs10795668	10p14	21	11
rs4444235	14q22.2/BMP4	21	9
rs4779584	15q13.3/GREM1	20	11

### The meta-analysis of 5 SNPs in associated with CRC risk

**Figure 3:**
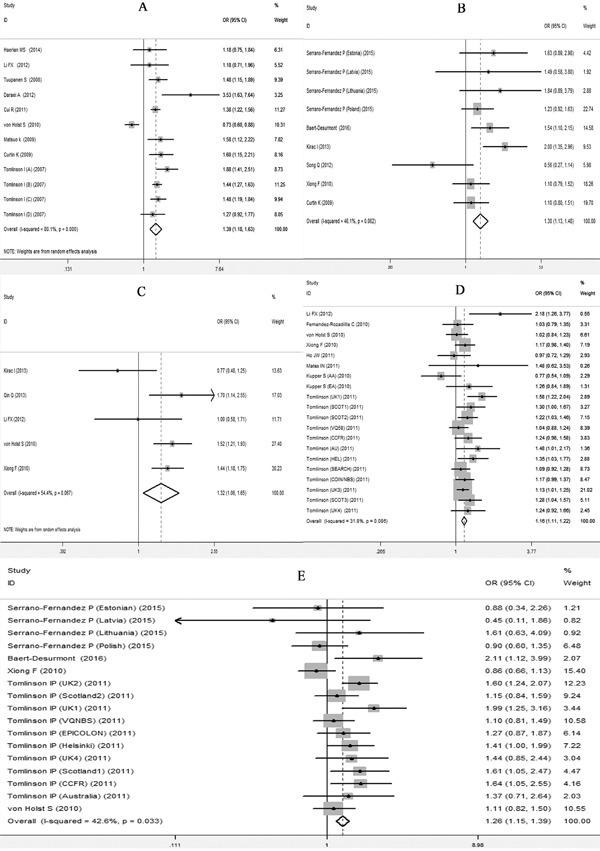
Forest plots for the 5 SNPs and risk of CRC in the homogenous model **A.** rs6938267, **B.** rs4939827, **C.** rs10795668, **D.** rs4444235, **E.** rs4779584.

The meta-analysis were performed based on two types of the data extracted from the included studies. One type was data with genotype counts (Supplementary Table S1), and the other type was that with OR and 95%CI values generated under various genetic models. Different genetic models were used in the meta-analysis. After pooling all of the included studies into the meta-analyses for each selected SNP, we found that all 5 SNPs were significantly associated with an increased risk of CRC (Table 4). Forest plots in the homogeneous genetic models are shown in Figure 3A-3E.

**Table 4:** Results of Meta-analysis for the selected 5 SNPs with CRC risk

SNPs	Source of Data	Heterogeneity			Meta-analysis results				
		x^2^	P-val	I^2^	Genetic model		OR/ES*	95% CI	P-val
rs6938267		55.15	<0.01	80.1%	Homogeneous GGvsTT	Random	1.388	1.180-1.633	<0.001
	Genotype Counts	24.77	0.01	55.6%	Heterogeneous GGvsGT	Random	1.154	1.051-1.267	0.003
		38.31	<0.01	71.3%	Dominant	Random	1.238	1.109-1.382	<0.001
		41.99	<0.01	73.8%	Recessive	Random	1.233	1.100-1.382	<0.001
		6.93	0.803	0.0%	log-additive	Fixed	1.170	1.126-1.213	<0.01
		4.85	0.678	0.0%	Allelic	Fixed	1.196	1.159-1.233	<0.001
	OR 95%CI	50.15	<0.01	90%	Homogeneous GGvsTT	Random	1.201	0.684-1.718	<0.001
		4.28	0.369	6.60%	Heterogeneous GGvsGT	Fixed	1.143	1.010-1.276	<0.001
rs4939827		14.48	0.062	46.1%	Homogeneous TTvsCC	Fixed	1.298	1.135-1.483	<0.001
	Genotype Counts	11.74	0.163	31.9%	Dominant	Fixed	1.269	1.168-1.380	<0.001
		15.14	0.052	48.2%	Recessive	Fixed	1.212	1.088-1.352	0.001
		56.98	<0.01	86.0%	log-additive	Random	1.066	0.948-1.183	<0.001
		25.76	<0.01	76.70%	Dominant	Random	1.263	0.938-1.588	<0.001
	OR 95%CI	13.84	0.008	71.10%	Homogeneous TTvsCC	Random	0.914	0.732-1.096	<0.001
		40.39	<0.01	87.60%	Heterogeneous TTvsTC	Random	1.074	0.877-1.270	<0.001
rs10795668		8.78	0.067	54.4%	Homogeneous GGvsAA	Random	1.323	1.062-1.648	0.012
	Genotype Counts	4.34	0.362	7.9%	Heterogeneous GGvsGA	Fixed	1.215	1.117-1.321	<0.001
		5.22	0.265	23.4%	Recessive	Fixed	1.248	1.153-1.351	<0.001
		29.81	<0.05	73.2%	log-additive	Random	1.021	0.930-1.111	<0.001
		7.49	0.112	46.6%	Allelic	Fixed	0.889	0.865-0.912	<0.001
	OR 95%CI	27.55	<0.05	81.9%	Homogeneous GGvsAA	Random	0.816	0.625-1.007	<0.001
		8.82	0.116	43.3%	Heterogeneous GGvsGA	Fixed	0.871	0.835-0.907	<0.001
rs4444235		27.87	0.086	31.8%	Homogeneous CCvsTT	Fixed	1.160	1.106-1.216	<0.001
	Genotype Counts	27.94	0.085	32.0%	Heterogeneous CCvsTC	Fixed	1.069	1.024-1.115	0.002
		21.4	0.315	11.20%	Dominant	Fixed	1.101	1.061-1.143	<0.001
		30.17	0.05	37.0%	Recessive	Fixed	1.100	1.056-1.145	<0.001
		8.77	0.362	8.8%	log-additive	Fixed	1.045	1.005-1.085	<0.001
		6.54	0.162	38.8%	Allelic	Fixed	1.043	0.983-1.102	<0.001
	OR 95%CI	4.27	0.234	29.8%	Homogeneous CCvsTT	Fixed	1.090	0.961-1.219	<0.001
		6.04	0.109	50.4%	Heterogeneous CCvsTC	Random	1.036	0.878-1.194	<0.001
rs4779584		27.86	0.033	42.6%	Homogeneous TTvsCC	Fixed	1.261	1.146-1.386	<0.001
	Genotype Counts	14.94	0.529	0.0%	Heterogeneous TTvsTC	Fixed	1.154	1.063-1.253	0.001
		28.25	0.030	43.4%	Dominant	Fixed	1.134	1.086-1.185	<0.001
		22.21	0.137	28.0%	Recessive	Fixed	1.189	1.100-1.286	<0.001
		2.42	0.788	0.0%	Dominant	Fixed	1.181	1.065-1.297	<0.001
		70.8	0.002	70.8%	Allelic	Random	1.270	1.149-1.390	<0.001
	OR 95%CI	4.64	0.200	35.4%	Homogeneous TTvsCC	Fixed	0.928	0.754-1.102	<0.001
		18.18	<0.05	83.5%	Heterogeneous TTvsTC	Random	0.995	0.728-1.262	<0.001

* ES (effect size) is the meta-analysis result from data source of OR 95%CI obtained in different genetic models.

#### rs6983267

The analysis of rs6983267 G allele carriers showed a strong association with CRC, with an OR of 1.388 (95% CI 1.180-1.8633; P<0.001) for the homogeneous model, an OR of 1.238 (95% CI 1.109-1.382; P<0.001) for the dominant model and an OR of 1.233 (95% CI 1.100-1.382; P<0.001) for the recessive model. These results were consistent with the risk estimates analyzed using OR values (95% CI, under homogenous model) collected from the included studies, with an effect size (ES) of 1.201 (ES>0; 95% CI 0.684-1.718; P<0.001).

The rs6983267 polymorphism is a G/T single-nucleotide variation on human chromosome 8p24. It has been associated with both prostate and CRC, and it was also the first SNP published to be associated with CRC risk [21]. Our meta-analysis showed a similar result to the previous study (OR=1.47 for GG homozygous). The SNP rs6983267 is located far away from any coding sequences. The nearest genes are the *MYC* oncogene and the putative pseudogene *POU5F1P1*, a transcription factor expressed in colon cancer cell lines [21, 31]. Berndt et al. discussed the potential contribution of *MYC* which is located > 300 KB distant to rs6983267 [32]. While, Tuupanen et al. didn't find clear association between rs6983267 genotype and *MYC* expression [33]. Thus, further studies are required as there still remains controversy between *MYC* and rs6983267.

The present meta-analysis was performed based on larger sample size and various source of data including 26,322 cases and 25,157 controls. The sensitivity analysis results showed that the significance of the pooled ORs was not influenced by any single study. However, an individual study von Holst S (2010) increased the heterogeneity (I^2^=71.3%, P=0.000) in homogeneous model across the studies included. The value of I^2^ in the same model reduced greatly (I^2^=14.3%, p=0.308) in the same genetic model after this study had been removed (Supplementary Figure S1A, S1B). The heterogeneity within the white subgroup also existed when stratified analysis was performed. The difference within the ethnicity may be due to the tumor location (proximal or distal distance) or/and stage. We couldn't have further analysis and discussion because the clinical and histopathologic characteristics were not provided by all studies.

#### rs4939827

The rs4939827 T allele had a significant association with CRC with an OR of 1.298 (95% CI 1.135-1.483, P<0.001) for the homogeneous model, an OR of 1.269 (95% CI 1.168-1.380; P<0.001) and an OR of 1.212 (95% CI 1.088-1.352; P<0.001) for the recessive model. The analysis on ORs (95% CI, under dominant model) in our meta-analysis showed a similar results with an ES of 1.263 (95% CI 0.938-1.588; P<0.001).

Variant rs4939827 is a C/T single-nucleotide variation located within intron 3 of *SMAD7* on chromosome 18q21. It has been associated with CRC. SMAD7 functions as an important pathway regulation step in the signaling of the TGF-β superfamily. The experiment demonstrates that some of the risk-associated alleles correlate with increased expression of SMAD7 in normal colon tissues, potentially perturb TGF-β negative feedback loop in TGF-β/BMP signaling pathways [34], thus make the progression of cell malignancy.

Broderick et al firstly identified rs4939827 in a GWA set of 620 cases and 960 controls and 3 replication sets of 7,377 cases and 5,867 controls [20], but in an opposite direction with an OR of 0.73 for CC homozygous. Multiple independent studies have replicated the association. The allele frequencies of this SNP in study von Holst S (2010) was found inconsistent with HWE. So it was removed from the present analysis for rs4939827 (data source of genotype counts). The results of present meta-analysis including 39,537 cases and 39,117 controls confirmed that the rs4939827 polymorphism is significantly associated with the increased CRC risk. Our results were similar to other independent studies [23, 35-37]. In addition, it is worth mentioning that our stratified analyses of ethnicities and regions indicate that the heterogeneity among the European descendants is higher than that among the Asians (Supplementary Figure S2).

#### rs10795668

In our study we found that rs10795668 A allele was significantly associated with CRC, having an OR of 1.323 (95% CI 1.062-1.648; P=0.012) for the homogeneous model, an OR of 1.248 (95% CI 1.153-1.351; P<0.001) for the recessive model. The analysis on ORs (95% CI, under homogeneous model) in our meta-analysis supported the risk estimate with an ES of 0.816 (ES>0; 95% CI 0.625-1.007; P<0.001).

The SNP rs10795668 with an A/G single-nucleotide variation maps to an 82-kb LD block (8.73–8.81 Mb) within 10p14 [22]. At present little is known about its functions. The SNP is located outside the coding regions of genes, and within 400 kb area around the SNP there are no known genes coding proteins. Loo LW et al. performed cis-expression quantitative trait loci analyses to investigate possible regulatory functions on the expression of neighboring genes [34]. They observed a significant association between the low CRC risk A allele for rs10795668 at 10p14 and increased expression of *ATP5C1*, which is a mitochondrial protein involved in cellular metabolism. Their findings suggested putative functional activities for the CRC GWAS identified risk loci as regulating the expression of neighboring genes.

The result of present meta-analysis including 37,294 cases and 41,037 controls also verified a significant association between the rs10795668 polymorphism and CRC risk, but in an opposite direction to the first report [22]. rs10795668 has been associated with CRC, yet with most conflicting results reported by multiple independent studies [5, 22, 24, 35, 36, 38-44]. The inconsistency in independent studies may be due to the limited sample size of some populations. In addition, rs10795668 exhibited a population difference among the European, African American and Asian populations [45], suggesting genetic heterogeneity across different ethnic groups or possible gene-environment interaction. Our stratified analysis also exhibited a population difference among different ethnicities. Therefore, further functional investigation is necessary to verify the study findings, which will help understand the mechanisms of the variant in the CRC development and tumor progression.

#### rs4444235

For SNP rs4444235, a significant association with CRC risk was confirmed with an OR equal to 1.160 (95% CI 1.106-1.216; P<0.001) for homogeneous model, 1.101 (95%CI 1.061-1.14; P<0.001) for the dominant and 1.100 (95% CI 1.056-1.145; P<0.001) for the recessive model. The effect size analyzed on homogeneous ORs source also showed similar effect (ES 1.090>0; 95%CI 0.961-1.219; P<0.001) to support the results.

SNP rs4444235 located 9.4 kb from the transcription start site of the gene encoding bone morphogenetic protein 4 preproprotein (BMP4), is a C/T single-nucleotide variation on human chromosome 14q22.2.

GWAS have identified rs4444235 as a new CRC and colorectal adenoma (CRA) susceptibility locus in populations of European descent. This hypothesis has been variously examined by a number of studies but with contradictory results [46-54]. A recent meta-analysis was performed with 54,631 CRC cases, 3,995 CRA cases and 88,098 controls from 15 studies [55]. In the stratified analysis on ethnicity, significantly increased risks were found in East Asians (OR = 1.07, 95% CI 1.01–1.12, *P* = 0.01) and Caucasians (OR = 1.07, 95% CI: 1.05–1.10, *P* < 10^−5^); while no significant associations were found among African Americans and other ethnic populations in all genetic models. The results implicated that rs4444235/BMP4 is a risk factor associated with increased CRC and CRA susceptibility, but these associations are vary in different ethnic groups.

The present meta-analysis including 33,024 CRC cases (according to inclusion criteria, CRA cases were excluded) and 33,348 controls showed that the rs4444235 polymorphism was significantly associated with increased risk of CRC under different genetic models.

#### rs4779584

Our analysis verified that rs4779584 was significantly associated with increased risk of CRC with an OR of 1.261 (95% CI 1.146-1.386; P<0.001) for the homogeneous model, an OR of 1.134 (1.086-1.185) and 1.189 (1.100-1.286) for the dominant and recessive model, respectively (P<0.001). The analysis on ORs (95% CI, under dominant model) in our meta-analysis showed a similar results with an ES of 1.181 (95% CI 1.065-1.297; P<0.001).

The rs4779584 polymorphism is a C/T single-nucleotide variation in the human genome on 15q13.3. It resides between *GREM1* and *SCG5. GREM1* is an important signaling molecule in the TGF-Δ pathway involved in tumor invasion and metastasis [56] acting as a bone morphogenetic protein (BMP) antagonist. Secretogranin V, encoded by SCG5 is an important neuroendocrine signaling molecule that appears to influence cellular proliferation in the large bowel based on nutrient availability or systemic hormonal effects. Several studies have reported association between this SNP and the risk of CRC. However, the results from the studies are inconclusive. Yang H et al. [57] conducted a meta-analysis of 12 independent case-control studies including 11,769 CRC cases and 14,328 healthy controls. The result showed that the rs4779584 polymorphism may increase the risk of developing CRC in Caucasian population.

The meta-analysis of this study included 33,008 cases of CRC and 35,715 controls. The analyses in various genetic models showed significant association between rs4779584 and increased risk of CRC. However, there was no significant difference among different ethnic populations.

The meta-analysis of this study was performed on 5 SNPs frequently reported by different research groups. The overall results showed that the selected 5 SNPs were strongly associated with CRC. The stratified analyses such as ethnicity and country or region suggested a difference among ethnicities, some even within the ethnicity. Cui R et al. [18] identified a novel susceptible locus at rs7758229/SLC22A3 on 6q26-q27 region that was significantly associated with distal colon cancer (OR 1.28; P=7.92× 10^−9^) in Asian population. This result suggests an ethnic diversity between Asians and Caucasians in the colorectal pathogenesis.

### Publication bias

**Figure 4:**
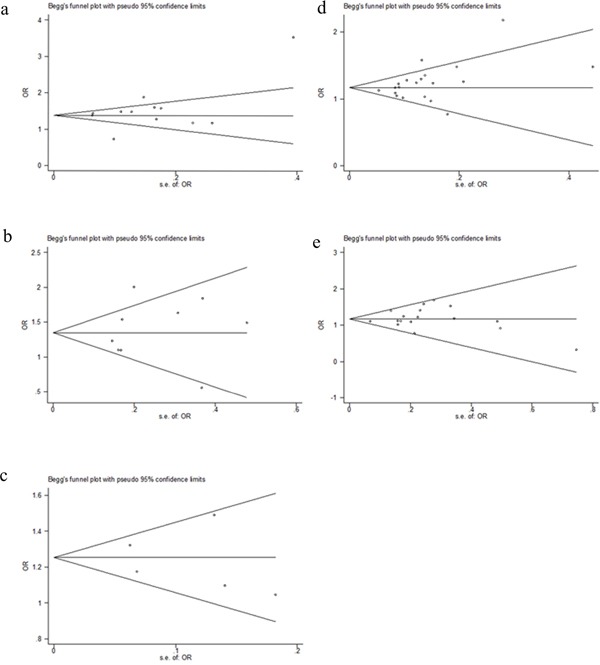
Begg's Funnel plot of 5 SNPs for Publication bias in the homogenous model **a.** rs6938267, **b.** rs4939827, **c.** rs10795668, **d.** rs4444235, **e.** rs4779584.

The Funnel plot and the Egger's test were used to assess publication bias. As showing in Figure 4a-4e, the shape of the funnel plots appeared to be symmetrical. The Egger's test did not detect any publication bias (data not shown).

### Sensitivity analyses

**Figure 5:**
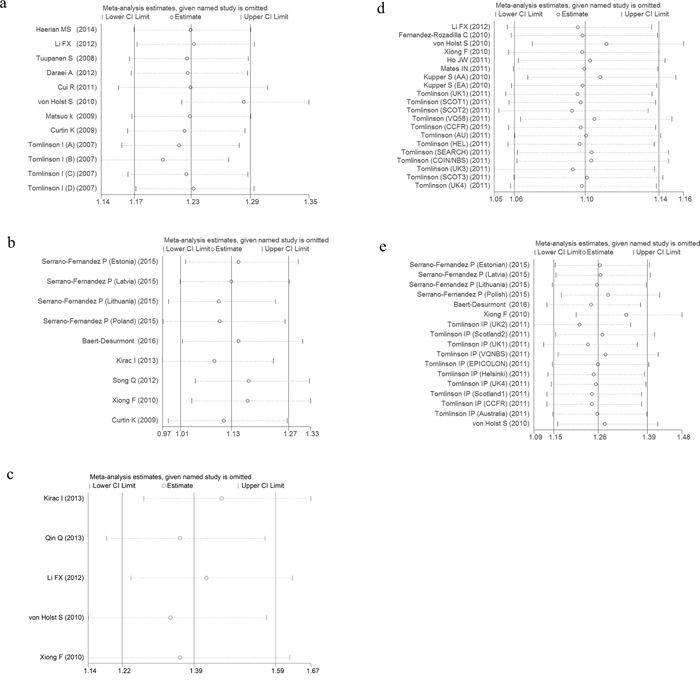
Sensitivity analysis of the odds ratio coefficients under dominant (a, c) and homogeneous (b, d, e) genetic models **a.** rs6938267, **b.** rs4939827, **c.** rs10795668, **d.** rs4444235, **e.** rs4779584.

Sensitivity analyses were performed after sequentially removing each eligible study. This approach is regarded as an indispensable step for analyzing multiple criteria. The significance of the pooled ORs was not influenced by any single study under dominant genetic model (Figure 5a-5e), indicating that our results were statistically robust and stable.

## CONCLUSION

In summary, this meta-analysis included a greater number of studies after SNP screening and publication inclusion. Therefore, a larger sample size (average 33,000 CRC cases and 34,000 controls per SNP) and increased statistical power were obtained. The analyses results of the selected 5 SNPs including rs6983267/8q24.21, rs4939827/18q21.1, rs10795668/10p14, rs44444235/ 14q22.2 and rs4779584/15q13.3 verified their associations with CRC risk. Moreover, the sensitivity analysis suggested no publication bias. The results are stable and reliable.

Early GWAS and replication studies mostly focused on the European and American white population. In comparison, the number of the studies on the Asians and African Americans is relatively small. However, the CRC incidence of these two populations are increasing. Different populations have common variants. While, the specific susceptibility loci might exist as well. The heterogeneity between different population groups was observed in the studies including our analysis. Therefore, further association studies on the Asian and African American population are needed to identify and verify common and unique CRC susceptibility loci in different populations. That will improve the understanding of the pathogenesis of CRC, lead to prognosis and therapeutic strategies and further help design potential CRC risk assessment model.

## MATERIALS AND METHODS

### Candidate SNPs screening

A literature searching was performed in GWAS central and PubMed without language restriction up to the end of 2014. The search strategy was based on combinations of the terms “GWAS, SNP” and “colorectal cancer or CRC”.

### Literature search

The most frequently reported SNPs were further searched in PubMed to end of June 2015. In addition, references in retrieved articles were scanned.

Studies were included if they met the all of the following criteria: (1) original study; (2) assessment of the association between the selected 5 SNPs and CRC risk; (3) case-control or cohort study design; (4) providing genotype numbers, odds ratio (OR) with corresponding confidence 95% interval (95%CI) or sufficient data to calculate them; (5) studies of humans; (6) the genotypes of the SNPs in controls are in HWE. We didn't included investigations in subjects with Lynch syndrome. Case reports, comments, reviews and editorials were also excluded. If the studies had overlapping subjects, only the study with the largest population was finally included. If more than one geographic or ethnic population were included in one report, each population was considered separately. All data were extracted independently by two reviewers and any disagreement was adjudicated by a third author.

### Data extraction

The following information was extracted from each study: first author's name, publication year, study country, ethnicity of study population, study type, genotyping method, numbers of cases and controls, control source, genotype numbers of the cases and controls or odds ratio (OR) and 95% confidence interval (CI).

### Statistical analyses

Hardy–Weinberg equilibrium (HWE) was tested by a goodness-of-fit χ^2^ test to compare the observed genotype frequencies to the expected genotype frequencies in controls (P<0.05).

To evaluate associations between SNP polymorphisms and risk of CRC, the pooled OR and associated 95% CI where calculated. We used the following models to calculate different ORs: the homogeneous and heterogeneous genetic models (AA vs. aa; Aa vs aa), the dominant genetic model (AA+Aa vs. aa), and the recessive genetic model (AA vs. Aa+aa).

Statistical heterogeneity across studies included was assessed by Cochran's Q-statistic and considered significant at P<0.05 [58]. The I^2^ statistic was then utilized to estimate heterogeneity quantitatively (I^2^= 0–25%, no heterogeneity; I^2^= 25–50%, moderate heterogeneity; I^2^= 50–75%, large heterogeneity; I^2^= 75–100%, extreme heterogeneity) [59]. The fixed effects model, by Mentel-Haenszel (M-H) method, was used to calculate the pooled estimate when I^2^<50; otherwise, the random-effects model, by DerSimonian-Laird (D+L) method, was applied when I^2^≥50 [60]. Sensitivity analysis was performed to assess the influence of each study on overall estimate by sequential removing of each study [61]. Publication bias was estimated by Begg's funnel plot and Eegger's test [62, 63]. All analyses were performed with the STATA Version 11.0 software (Stata Corp, College Station, TX). All P values in this study were two-tailed tested with a significant level at 0.05.

## SUPPLEMENTARY MATERIALS FIGURES AND TABLES




