# Supplementary material for: A comprehensive meta-analysis of genetic associations between five key SNPs and colorectal cancer risk
Source: Oncotarget. 2016 Sep 21;7(45):73945–59. doi: 10.18632/oncotarget.12154 (PMC5342026; doi:10.18632/oncotarget.12154)
Supplement: Supplementary file 1 [file oncotarget-07-73945-s001.pdf]

A comprehensive meta-analysis of genetic associations between five key SNPs and colorectal cancer risk

SUPPLEMENTARY FIGURES AND TABLES

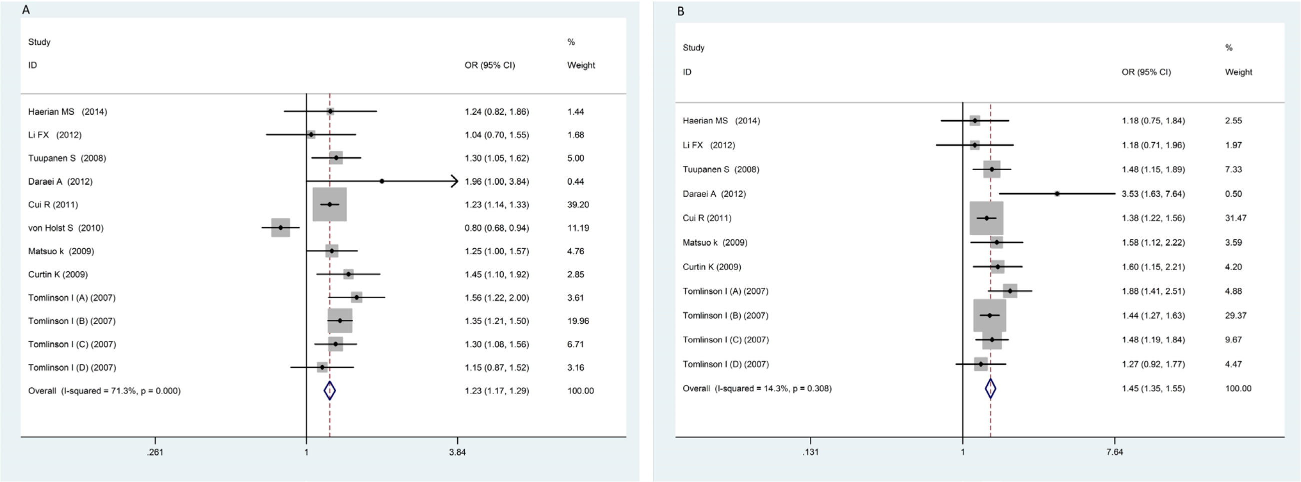

Supplementary Figure S1: Forest plot for rs6983267 and risk of CRC in homogenous model. A. with the study von Holst S (2010) having I^2=71.3%, P=0.000; **B.** without the study von Holst S (2010) having I^2=14.3%, P=0.308.

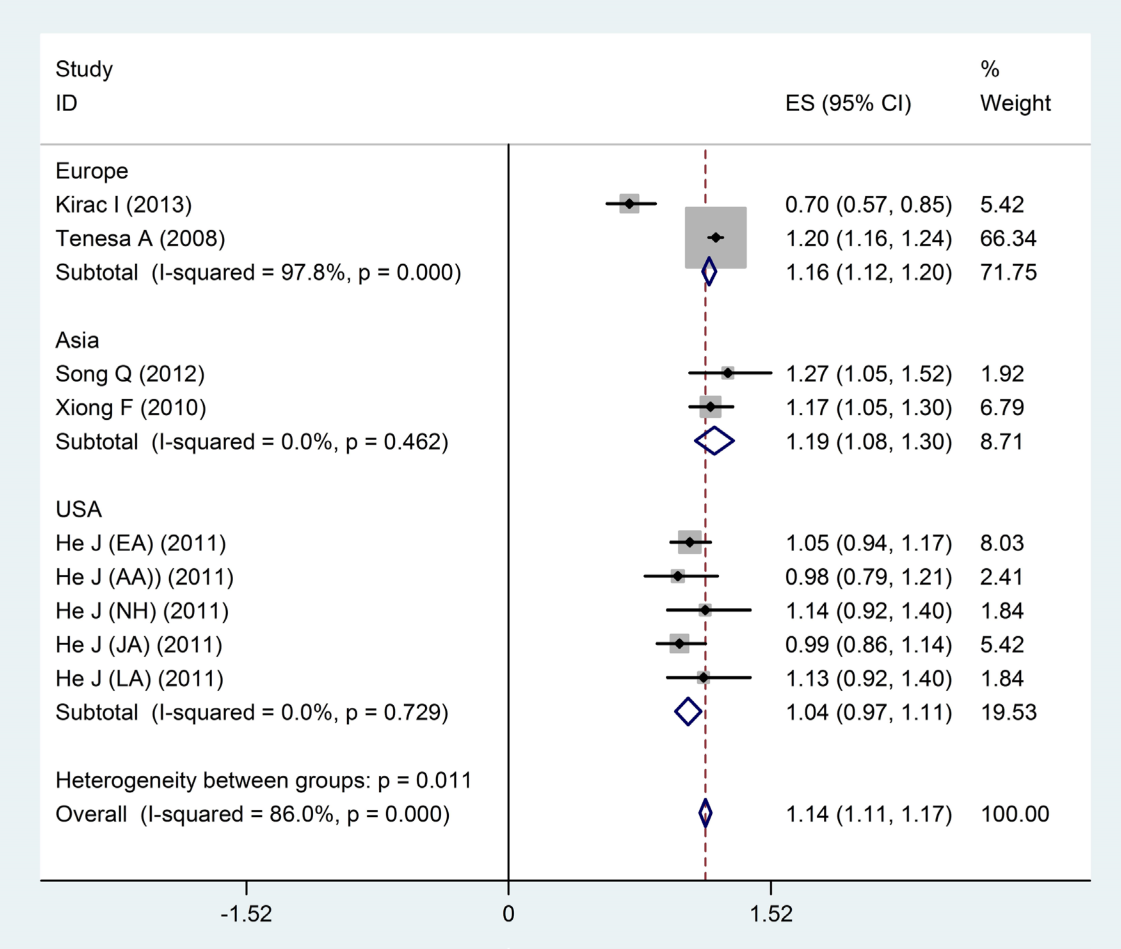

Supplementary Figure S2: Stratified analyses of rs4939827 on ethnicities and regions with CRC risk indicating that the heterogeneity among the European descendants is higher than that among the Asians.

Supplementary Table S1: Genotype No. of selected 5SNPs and p-values of HWE calculation

See Supplementary File 1

Supplementary Table S2: Characteristics of studies on the association between rs4939827 and CRC risk included in the meta-analysis

First author	Year	Ethnicity (Population)	Number	
			Case	Control
Serrano-Fernandez P	2015	Estonian	166	166
Serrano-Fernandez P	2015	Latvia	81	81
Serrano-Fernandez P	2015	Lithuania	123	123
Serrano-Fernandez P	2015	Poland	795	795
Baert-Desurmont	2016	French	1029	350
Zhang B	2014	East Asians	1773	2642
Kirac I	2013	Croatian	320	954
Song Q	2012	Chinese	641	1037
He J	2011	European Americans	1171	1534
He J	2011	African Americans	382	510
He J	2011	Native Hawaiians	323	472
He J	2011	Japanese Americans	1042	1246
He J	2011	Latinos	393	524
von Holst S	2010	Swedish	1786	1749
Xiong F	2010	Chinese	2124	2124
NiittymÃ¤ki I	2010	Fennish	970	969
Curtin K	2009	UK	654	621
Tenesa A	2008	Scotland	17457	16353
Broderick P	2007	UK	8307	6867

Supplementary Table S3: Characteristics of studies on the association between rs10795668 and CRC risk included in the meta-analysis

First author	Year	Ethnicity (Population)	Number	
			Case	Control
Tomlinson IP	2008	Mixed White (UK; The Netherlands; Finnish; Spanish; Australian; German)	18831	18540
Peters U	2012	Mixed White (USA, Canada, Australia, Germany, Israel, France)	7681	8790
Yang CY	2014	Taiwan Chinese	705	1802
Kirac I	2013	Croatian	320	594
Qin Q	2013	Chinese	470	475
Li FX	2012	Chinese	229	267
Ho JW	2011	Hong Kong Chinese	892	890
He J	2011	European American	1171	1534
He J	2011	African American	382	510
He J	2011	Native Hawaiian	323	472
He J	2011	Japanese American	1042	1426
He J	2011	Latino	393	524
von Holst S	2010	Swedish	1786	1749
Xiong F	2010	Chinese	2124	2124
Middeldorp A	2009	Dutch	945	1340

Supplementary Table S4: Characteristics of studies on the association between rs4444235 and CRC risk included in the meta-analysis

First author	Year	Ethnicity (Population)	Number	
			Case	Control
Li FX	2012	Chinese	229	267
Femandez-Rozadilla C	2010	Spainish	854	892
von Holst S	2010	Swedish	1758	1697
Xiong F	2010	Chinese	2124	2124
Ho JW	2011	Hongkong Chinese	892	890
Mates IN	2011	Romanian	92	96
Kupper S	2010	African American	795	985
Kupper S	2010	European American	399	367
He J	2011	European American	1171	1534
He J	2011	African American	382	510
He J	2011	Native Hawaiian	323	472
He J	2011	Japanese American	1042	1426
He J	2011	Latino	393	524
Tomlinson	2011	UK	922	929
Tomlinson	2011	Scotland	980	1002
Tomlinson	2011	Scotland	2024	2092
Tomlinson	2011	England, Wales and Scotland	1832	2720
Tomlinson	2011	North American, Australian	1332	1084
Tomlinson	2011	Australian	441	441
Tomlinson	2011	Finland	988	864
Tomlinson	2011	UK	2248	2209
Tomlinson	2011	COIN/NBS	2125	2501
Tomlinson	2011	UK	7912	4398
Tomlinson	2011	Scotland	1145	2203
Tomlinson	2011	UK	621	1121

Supplementary Table S5: Characteristics of studies on the association between rs4779584 and CRC risk included in the meta-analysis

First author	Year	Ethnicity (Population)	Number	
			Case	Control
Serrano-Fernandez P	2015	Estonian	166	166
Serrano-Fernandez P	2015	Latvian	81	81
Serrano-Fernandez P	2015	Lithuanian	123	123
Serrano-Fernandez P	2015	Polish	795	795
Baert-Desurmont	2016	French	1029	350
Kupfer SS	2014	African American	795	985
Kupfer SS	2014	European American	399	367
Xiong F	2010	Chinese	2124	2124
Houlston RS	2008	London phase1	940	965
Houlston RS	2008	Edinburgh phase1	1012	1012
Houlston RS	2008	Lodon phase2	2873	1235
Houlston RS	2008	Edinburgh phase2	2057	2111
Hes FJ	2014	Netherland	252	745
Tomlinson IP	2011	UK2	2854	2822
Tomlinson IP	2011	Scotland 2	2024	2082
Tomlinson IP	2011	UK1	922	929
Tomlinson IP	2011	UK(England, Wales and Scotland)	1832	2720
Tomlinson IP	2011	EPICOLON (Spain)	1410	1410
Tomlinson IP	2011	Finnish	988	864
Tomlinson IP	2011	UK4	621	1121
Tomlinson IP	2011	Scotland1	980	1002
Tomlinson IP	2011	Mixed (North American, Australian)	1332	1084
Tomlinson IP	2011	Australia	591	2353
Ho JW	2011	Chinese	716	714
He J	2011	European American	1171	1534
He J	2011	African American	382	510
He J	2011	Native Hawaiian	323	472
He J	2011	Japanese American	1042	1426
He J	2011	Latino	393	524
von Holst S	2010	Swedish	1786	1749
Middeldorp A	2009	Netherland	995	1340
