# Supplementary material for: A comprehensive meta-analysis of genetic associations between five key SNPs and colorectal cancer risk
Source: Oncotarget. 2016 Sep 21;7(45):73945–59. doi: 10.18632/oncotarget.12154 (PMC5342026; doi:10.18632/oncotarget.12154)
Supplement: Supplementary file 2 [file oncotarget-07-73945-s002.docx]

## **Supplementary tables**

**Table S1. Genotype No. of selected 5SNPs and p-values of HWE calculation**.

| **SNP** | **First author** | **Year**  **published** | **Country** | **Ethnicity**  **group** | **Cases** | **Controls** | **Case genotype no.** | | | **Control genotype no.** | | | **HWE**  **p-val** |
| --- | --- | --- | --- | --- | --- | --- | --- | --- | --- | --- | --- | --- | --- |
|  |  |  |  |  |  |  | 11 | 12 | 22 | 11 | 12 | 22 |  |
| rs6983267 | Haerian MS | 2014 | Iran | Caucasian | 380 | 335 | 53 | 190 | 137 | 56 | 156 | 123 | 0.5848 |
|  | Li FX | 2012 | China | Asian | 229 | 267 | 71 | 100 | 53 | 74 | 106 | 47 | 0.4274 |
|  | Tuupanen S | 2008 | Finland | Caucasian | 996 | 1012 | 185 | 478 | 333 | 232 | 497 | 283 | 0.6268 |
|  | Daraei A | 2012 | Iran | Caucasian | 115 | 120 | 16 | 45 | 49 | 30 | 64 | 26 | 0.4573 |
|  | Cui R | 2011 | Japan | Asian | 6167 | 4494 | 2347 | 2934 | 880 | 1937 | 2029 | 528 | 0.9241 |
|  | von Holst S | 2010 | Sweden | Caucasian | 1786 | 1749 | 397 | 890 | 450 | 332 | 892 | 517 | 0.1288 |
|  | Matsuo k | 2009 | Japan | Asian | 481 | 962 | 181 | 222 | 73 | 418 | 436 | 107 | 0.6748 |
|  | Curtin K | 2009 | UK | Caucasian | 654 | 621 | 109 | 329 | 205 | 140 | 307 | 165 | 0.9026 |
|  | Tomlinson I (A) | 2007 | UK | Caucasian | 620 | 960 | 116 | 302 | 202 | 254 | 471 | 235 | 0.5693 |
|  | Tomlinson I (B) | 2007 | UK | Caucasian | 4361 | 3752 | 811 | 2216 | 1334 | 883 | 1857 | 1012 | 0.5833 |
|  | Tomlinson I (C) | 2007 | UK | Caucasian | 1901 | 1079 | 342 | 943 | 616 | 239 | 549 | 291 | 0.5117 |
|  | Tomlinson I (D) | 2007 | UK | Caucasian | 1072 | 415 | 208 | 549 | 315 | 90 | 218 | 107 | 0.2861 |

| rs4939827 | Serrano-Fernandez P (Estonia) | 2015 | Estonia | Caucasian | 166 | 166 | 32 | 87 | 47 | 50 | 71 | 45 | 0.0639 |
| --- | --- | --- | --- | --- | --- | --- | --- | --- | --- | --- | --- | --- | --- |
|  | Serrano-Fernandez P (Latvia) | 2015 | Latvia | Caucasian | 81 | 81 | 15 | 46 | 20 | 19 | 45 | 17 | 0.3144 |
|  | Serrano-Fernandez P (Lithuania) | 2015 | Lithuania | Caucasian | 123 | 123 | 25 | 52 | 46 | 27 | 69 | 27 | 0.1726 |
|  | Serrano-Fernandez P (Poland) | 2015 | Poland | Caucasian | 795 | 795 | 157 | 393 | 245 | 167 | 416 | 212 | 0.1594 |
|  | Baert-Desurmont | 2016 | France | Caucasian | 1029 | 350 | 191 | 493 | 343 | 89 | 157 | 104 | 0.0583 |
|  | Kirac I | 2013 | Croatia | Caucasian | 320 | 954 | 63 | 143 | 96 | 172 | 291 | 131 | 0.7054 |
|  | Song Q | 2012 | China | Asian | 641 | 1037 | 399 | 232 | 10 | 732 | 272 | 33 | 0.214 |
|  | von Holst S | 2010 | Sweden | Caucasian | 1786 | 1749 | 395 | 886 | 501 | 387 | 884 | 408 | 0.0293 |
|  | Xiong F | 2010 | China | Asian | 2124 | 2124 | 1370 | 677 | 77 | 1442 | 570 | 74 | 0.0605 |
|  | Curtin K | 2009 | UK | Caucasian | 654 | 621 | 134 | 301 | 208 | 126 | 312 | 178 | 0.6151 |

**Table S1. Genotype No. of selected 5SNPs and p-values of HWE calculation. (Continued)**

| **SNP** | **First author** | **Year**  **published** | **Country** | **Ethnicity**  **group** | **Cases** | **Controls** | **Case genotype no.** | | | | | **Control genotype no.** | | | | | | **HWE**  **p-val** |
| --- | --- | --- | --- | --- | --- | --- | --- | --- | --- | --- | --- | --- | --- | --- | --- | --- | --- | --- |
|  |  |  |  |  |  |  | **11** | **12** | | **22** | | **11** | | | **12** | **22** | |  |
| rs10795668 | Kirac I | 2013 | 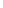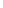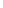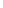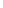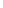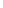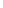   \| Croatia \| \| --- \| | Caucasian | 320 | 594 | 34 | 128 | | 148 | | 48 | | | 276 | 270 | | 0.0507 |
|  | Qin Q | 2013 | China | Asian | 470 | 475 | 53 | 187 | | 230 | | 73 | | | 216 | 186 | | 0.4331 |
|  | Li FX | 2012 | China | Asian | 229 | 267 | 32 | 85 | | 106 | | 37 | | | 105 | 123 | | 0.0629 |
|  | von Holst S | 2010 | Sweden | Caucasian | 1786 | 1749 | 148 | 779 | | 853 | | 197 | | | 754 | 745 | | 0.7669 |
|  | Xiong F | 2010 | China | Asian | 2124 | 2124 | 232 | 910 | | 963 | | 286 | | | 1010 | 827 | | 0.4184 |
| rs4444235 | **Li FX** | 2012 | China | Asian | 229 | 267 | 35 | | 122 | | 58 | | 71 | 144 | | 54 | 0.2191 | |
|  | Femandez-Rozadilla C | 2010 | Spain | Caucasian | 854 | 892 | 168 | | 436 | | 242 | | 196 | 411 | | 274 | 0.0769 | |
|  | von Holst S | 2010 | Sweden | Caucasian | 1758 | 1697 | 573 | | 829 | | 356 | | 533 | 838 | | 326 | 0.9166 | |
|  | Xiong F | 2010 | China | Asian | 2124 | 2124 | 583 | | 1091 | | 427 | | 639 | 1085 | | 399 | 0.1032 | |
|  | Ho JW | 2011 | Hongkong | Asian | 892 | 890 | 170 | | 350 | | 195 | | 168 | 346 | | 199 | 0.4609 | |
|  | Mates IN | 2011 | Romania | Caucasian | 92 | 96 | 18 | | 52 | | 22 | | 23 | 54 | | 19 | 0.2135 | |
|  | Kupper S (AA) | 2010 | US-AA | African | 795 | 985 | 332 | | 319 | | 62 | | 400 | 418 | | 97 | 0.4282 | |
|  | Kupper S (EA) | 2010 | US-EA | Caucasian | 399 | 367 | 93 | | 183 | | 97 | | 100 | 163 | | 83 | 0.3017 | |
|  | Tomlinson (UK1) | 2011 | UK | Caucasian | 922 | 929 | 233 | | 441 | | 247 | | 274 | 470 | | 184 | 0.4921 | |
|  | Tomlinson (SCOT1) | 2011 | UK | Caucasian | 980 | 1002 | 256 | | 500 | | 220 | | 294 | 512 | | 195 | 0.2953 | |
|  | Tomlinson (SCOT2) | 2011 | UK | Caucasian | 2024 | 2092 | 540 | | 1017 | | 449 | | 630 | 999 | | 428 | 0.3833 | |
|  | Tomlinson (VQ58) | 2011 | UK | Caucasian | 1832 | 2720 | 503 | | 886 | | 410 | | 773 | 1312 | | 603 | 0.3025 | |
|  | Tomlinson (CCFR) | 2011 | UK | Caucasian | 1332 | 1084 | 290 | | 595 | | 298 | | 274 | 496 | | 227 | 0.9296 | |
|  | Tomlinson (AU) | 2011 | UK | Caucasian | 441 | 441 | 124 | | 208 | | 108 | | 129 | 233 | | 76 | 0.0952 | |
|  | Tomlinson (HEL) | 2011 | UK | Caucasian | 988 | 864 | 272 | | 459 | | 202 | | 273 | 405 | | 150 | 0.9923 | |
|  | Tomlinson (SEARCH) | 2011 | UK | Caucasian | 2248 | 2209 | 618 | | 1083 | | 537 | | 650 | 1086 | | 519 | 0.1112 | |
|  | Tomlinson (COIN/NBS) | 2011 | UK | Caucasian | 2125 | 2501 | 593 | | 1044 | | 510 | | 722 | 1246 | | 532 | 0.8969 | |
|  | Tomlinson (UK3) | 2011 | UK | Caucasian | 7912 | 4398 | 2012 | | 3865 | | 1828 | | 1247 | 2116 | | 1006 | 0.0605 | |
|  | Tomlinson (SCOT3) | 2011 | UK | Caucasian | 1145 | 2203 | 305 | | 554 | | 268 | | 628 | 1130 | | 432 | 0.0593 | |
|  | Tomlinson (UK4) | 2011 | UK | Caucasian | 621 | 1121 | 141 | | 306 | | 127 | | 288 | 544 | | 210 | 0.1063 | |

**Table S1. Genotype No. of selected 5SNPs and p-values of HWE calculation. (Continued)**

| **SNP** | **First author** | **Year**  **published** | **Country** | **Ethnicity**  **group** | **Cases** | **Controls** | **Case genotype no.*** | | | **Control genotype no.** | | | **HWE** |
| --- | --- | --- | --- | --- | --- | --- | --- | --- | --- | --- | --- | --- | --- |
|  |  |  |  |  |  |  | **11** | **12** | **22** | **11** | **12** | **22** | **p-val** |
| rs4779584 | Serrano-Fernandez P (Estonian) | 2015 | Estonian | Caucasian | 166 | 166 | 99 | 58 | 9 | 97 | 59 | 10 | 0.797 |
|  | Serrano-Fernandez P (Latvia) | 2015 | Latvia | Caucasian | 81 | 81 | 49 | 29 | 3 | 52 | 22 | 7 | 0.0538 |
|  | Serrano-Fernandez P (Lithuania) | 2015 | Lithuania | Caucasian | 123 | 123 | 58 | 53 | 12 | 70 | 44 | 9 | 0.5702 |
|  | Serrano-Fernandez P (Polish) | 2015 | Poland | Caucasian | 795 | 795 | 446 | 301 | 48 | 467 | 272 | 56 | 0.0622 |
|  | Baert-Desurmont | 2016 | French | Caucasian | 1029 | 350 | 563 | 402 | 64 | 223 | 115 | 12 | 0.5453 |
|  | Xiong F | 2010 | China | Caucasian | 2124 | 2124 | 128 | 627 | 1353 | 109 | 682 | 1333 | 0.0759 |
|  | Tomlinson IP (UK2) | 2011 | UK | Asian | 2854 | 2822 | 1762 | 934 | 155 | 1858 | 857 | 102 | 0.7962 |
|  | Tomlinson IP (Scotland2) | 2011 | UK | Caucasian | 2024 | 2082 | 1276 | 603 | 84 | 1332 | 608 | 76 | 0.5243 |
|  | Tomlinson IP (UK1) | 2011 | UK | Caucasian | 922 | 929 | 533 | 316 | 52 | 611 | 288 | 30 | 0.5767 |
|  | Tomlinson IP (VQNBS) | 2011 | UK | Caucasian | 1832 | 2720 | 1155 | 564 | 81 | 1601 | 797 | 102 | 0.8221 |
|  | Tomlinson IP (EPICOLON) | 2011 | Spain | Caucasian | 1410 | 1410 | 878 | 434 | 61 | 934 | 396 | 51 | 0.2664 |
|  | Tomlinson IP (Helsinki) | 2011 | Finland | Caucasian | 988 | 864 | 378 | 362 | 88 | 418 | 352 | 69 | 0.671 |
|  | Tomlinson IP (UK4) | 2011 | UK | Caucasian | 621 | 1121 | 361 | 174 | 33 | 426 | 241 | 27 | 0.3239 |
|  | Tomlinson IP (Scotland1) | 2011 | UK | Caucasian | 980 | 1002 | 591 | 331 | 55 | 676 | 286 | 39 | 0.2093 |
|  | Tomlinson IP (CCFR) | 2011 | North America, Australia | Caucasian | 1332 | 1084 | 716 | 412 | 58 | 647 | 319 | 32 | 0.3327 |
|  | Tomlinson IP (Australia) | 2011 | Australia | Caucasian | 591 | 2353 | 269 | 149 | 22 | 285 | 136 | 17 | 0.8775 |
|  | von Holst S | 2010 | Sweden | Caucasian | 1786 | 1749 | 1050 | 572 | 94 | 1104 | 551 | 89 | 0.0634 |

*11,12,22 representing genotypes. rs6983267: TT,GT,GG ; rs4939827:CC, TC,TT; rs4779584: AA, AG,GG; rs4444235: TT,TC,CC; rs4779584:CC,TC,TT

HWE: Hardy–Weinberg equilibrium
